# Multiple Transporters and Glycoside Hydrolases Are Involved in Arabinoxylan-Derived Oligosaccharide Utilization in Bifidobacterium pseudocatenulatum

**DOI:** 10.1128/AEM.01782-20

**Published:** 2020-11-24

**Authors:** Yuki Saito, Akira Shigehisa, Yohei Watanabe, Naoki Tsukuda, Kaoru Moriyama-Ohara, Taeko Hara, Satoshi Matsumoto, Hirokazu Tsuji, Takahiro Matsuki

**Affiliations:** a Yakult Central Institute, Tokyo, Japan; b Yakult Honsha European Research Center for Microbiology, ESV, Ghent, Belgium; Shanghai Jiao Tong University

**Keywords:** ABC transporters, GH43, arabinoxylan, bifidobacteria, dietary fiber, glycoside hydrolase, oligosaccharides

## Abstract

Arabinoxylan hydrolysates (AXH) are the hydrolyzed products of the major components of the dietary fiber arabinoxylan. AXH include diverse oligosaccharides varying in xylose polymerization and side residue modifications with arabinose at the O-2 and/or O-3 position of the xylose unit. Previous studies have reported that AXH exhibit prebiotic properties on gut bifidobacteria; moreover, several adult-associated bifidobacterial species (e.g., Bifidobacterium adolescentis and Bifidobacterium longum subsp. *longum*) are known to utilize AXH. In this study, we tried to elucidate the molecular mechanisms of AXH utilization by Bifidobacterium pseudocatenulatum, which is a common bifidobacterial species found in adult feces. We performed transcriptomic analysis of B. pseudocatenulatum YIT 4072^T^, which identified three upregulated gene clusters during AXH utilization. The gene clusters encoded three sets of ATP-binding cassette (ABC) transporters and five enzymes belonging to glycoside hydrolase family 43 (GH43). By characterizing the recombinant proteins, we found that three solute-binding proteins of ABC transporters showed either broad or narrow specificity, two arabinofuranosidases hydrolyzed either single- or double-decorated arabinoxylooligosaccharides, and three xylosidases exhibited functionally identical activity. These data collectively suggest that the transporters and glycoside hydrolases, encoded in the three gene clusters, work together to utilize AXH of different sizes and with different side residue modifications. Thus, our study sheds light on the overall picture of how these proteins collaborate for the utilization of AXH in B. pseudocatenulatum and may explain the predominance of this symbiont species in the adult human gut.

**IMPORTANCE** Bifidobacteria commonly reside in the human intestine and possess abundant genes involved in carbohydrate utilization. Arabinoxylan hydrolysates (AXH) are hydrolyzed products of arabinoxylan, one of the most abundant dietary fibers, and they include xylooligosaccharides and those decorated with arabinofuranosyl residues. The molecular mechanism by which B. pseudocatenulatum, a common bifidobacterial species found in adult feces, utilizes structurally and compositionally variable AXH has yet to be extensively investigated. In this study, we identified three gene clusters (encoding five GH43 enzymes and three solute-binding proteins of ABC transporters) that were upregulated in B. pseudocatenulatum YIT 4072^T^ during AXH utilization. By investigating their substrate specificities, we revealed how these proteins are involved in the uptake and degradation of AXH. These molecular insights may provide a better understanding of how resident bifidobacteria colonize the colon.

## Abstract (precis)

Bifidobacteria commonly reside in the human intestine and possess abundant genes involved in carbohydrate utilization. Arabinoxylan hydrolysates (AXH) are hydrolyzed products of arabinoxylan, one of the most abundant dietary fibers, and they include xylooligosaccharides and those decorated with arabinofuranosyl residues. The molecular mechanism by which B. pseudocatenulatum, a common bifidobacterial species found in adult feces, utilizes structurally and compositionally variable AXH has yet to be extensively investigated. In this study, we identified three gene clusters (encoding five GH43 enzymes and three solute-binding proteins of ABC transporters) that were upregulated in B. pseudocatenulatum YIT 4072^T^ during AXH utilization. By investigating their substrate specificities, we revealed how these proteins are involved in the uptake and degradation of AXH. These molecular insights may provide a better understanding of how resident bifidobacteria colonize the colon.

## INTRODUCTION

The gastrointestinal tract is inhabited by diverse bacteria. Dietary fiber is the main carbon source for these resident bacteria and affects the composition of the gut microbiota (1). The human gut microbiota possesses various carbohydrate-active enzymes (CAZymes) that can degrade plant-derived polysaccharides (2).

Bifidobacteria are commonly found in the human gut throughout life; however, their composition differs between infancy and adulthood. Bifidobacteria exhibit a saccharolytic feature, which contributes to their adaptation to the environment (3). The infant gut microbiota is dominated by several bifidobacterial species (e.g., Bifidobacterium longum subsp. *infantis*, Bifidobacterium bifidum, and Bifidobacterium breve) that are known to utilize various human milk oligosaccharides (4–7). In the adult gut microbiota, other bifidobacterial species (e.g., Bifidobacterium pseudocatenulatum, Bifidobacterium adolescentis, and Bifidobacterium longum subsp. *longum*) are predominant (8, 9). Genomic analysis and culture-based assays suggest that the last two taxa are able to utilize plant-derived carbohydrates (10–12). Thus, plant-derived carbohydrates could be a factor driving bifidobacterial colonization in the adult gut.

Arabinoxylan (AX) is a major hemicellulose constituent of the plant cell wall and comprises long chains of β-1,4-linked xylose partially decorated with α-l-arabinofuranosyl residues at the O-2 and/or O-3 position (13). Xylanolytic bacteria, such as *Bacteroides* strains, primarily hydrolyze AX into arabinoxylan hydrolysates (AXH; also referred as arabinoxylan-oligosaccharides [AX-OS]) by an extracellular endo-xylanase and supply oligosaccharides to bifidobacteria (14). The AXH include both xylooligosaccharides (XOS) and arabinoxylooligosaccharides (AXOS). Several human and animal studies have shown that the intake of an AXH-rich diet resulted in an increased abundance of the genus *Bifidobacterium* (15–17). It has been reported that several adult-associated bifidobacterial strains (e.g., Bifidobacterium adolescentis and Bifidobacterium longum subsp. *longum*) assimilate AXH and possess enzymes involved in the degradation of AXH (18–21). Bifidobacterium pseudocatenulatum is a common bifidobacterial species found in adult feces. However, molecular insights into how B. pseudocatenulatum utilizes structurally diverse AXH are lacking.

In this study, we investigated the molecular mechanism of bifidobacterial AXH utilization using B. pseudocatenulatum YIT 4072^T^ (= JCM 1200^T^). On performing a comprehensive transcriptomic analysis, we found that the genes for ABC transporters and glycoside hydrolases were upregulated during AXH utilization. We further investigated the substrate specificities of these proteins to shed light on how B. pseudocatenulatum utilizes the AXH, which include oligosaccharides varying in the length of polymerization and the position of the arabinofuranosyl decoration.

## RESULTS

### Growth of B. pseudocatenulatum on AX-related carbohydrates.

**FIG 1:**
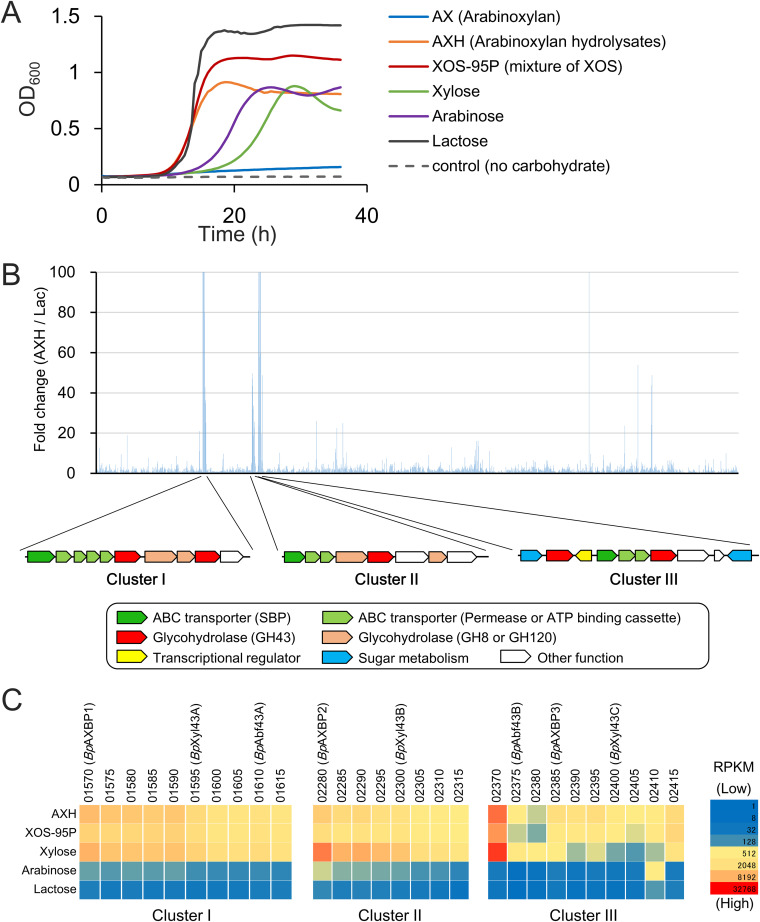
Gene clusters responsible for utilizing AXH. (A) Growth curves of B. pseudocatenulatum YIT 4072^T^ on AX-related carbohydrates. (B) (Top) Ratio of the RPKM value of B. pseudocatenulatum YIT 4072^T^ grown on the different carbon sources. The ratios of the RPKM values (AXH/lactose [Lac]) are aligned in the order of the gene locus. For genes with an RPKM value of zero under the lactose condition, the ratio was calculated with the number of reads set equal to 1. The vertical axis shows the part with values below 100. (Bottom) The gene organization of the three clusters which displayed high levels expression during growth on the AXH are shown. (C) The RPKM values of genes encoded by the three clusters are represented by a heat map. The numbers represent abbreviated locus tags (BBPC_RSXXXXX).

To evaluate the ability of B. pseudocatenulatum YIT 4072^T^ to utilize AX-related carbohydrates, we cultured the strain using media containing AX, AXH (see Materials and Methods for a description of its preparation), a commercially available mixture of XOS (XOS-95P; provided by B Food Science Co., Ltd., Aichi, Japan), xylose, or arabinose as the sole carbon source (final concentration, 0.5%). We found the more rapid growth of the strain in the presence of AX-derived oligosaccharides than in the presence of AX-derived monosaccharides: the lag time of AXH and XOS-95P (10.5 h) was shorter than that of xylose and arabinose (17.5 and 14.5 h, respectively), and the generation time of AXH and XOS-95P (110 and 100 min, respectively) was shorter than that of xylose and arabinose (210 and 170 min, respectively) (Fig. 1A). The growth of the strain was not observed in the presence of AX or the control.

### Transcriptomic analysis during growth on media supplemented with AXH.

We subsequently performed transcriptomic analysis (RNA-seq) to find the genes that were involved in AXH utilization (Fig. 1B). We found that 10 genes (BBPC_RS01570 to BBPC_RS01615) were located in a cluster (denoted cluster I; Fig. 1B) and upregulated during growth with AXH (upregulation at a level 10 times greater than that of lactose; number of reads per kilobase per million reads [RPKM] > 500; Fig. 1B and C). In addition, we found that the other upregulated genes (BBPC_RS02280 to BBPC_RS02315 and BBPC_RS02370 to BBPC_RS02415) were encoded in two clusters (denoted clusters II and III, respectively) (Fig. 1B and C). Referring to the annotation, the genes located in these three clusters were predicted to be associated with carbohydrate utilization. The upregulated genes in these clusters included three sets of ABC transporters and nine glycoside hydrolases (GH) which belonged to GH family 43 (GH43), GH8, or GH120 (see Fig. S1 in the supplemental material). We observed that these clusters were also upregulated with xylose and XOS-95P but not with arabinose (Fig. 1C). All nine GH enzymes lacked a signal peptide, suggesting that the enzyme hydrolyzes the substrate intracellularly.

### Substrate specificities of SBP.

**FIG 2:**
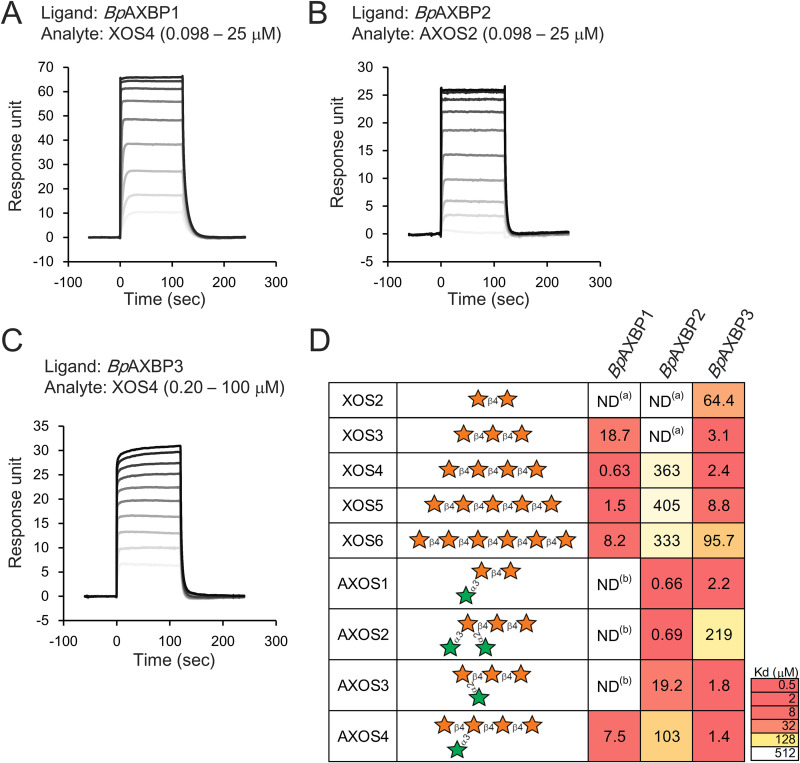
Affinities of the *Bp*AXBP for the differently decorated AXOS or XOS with different polymerizations. (A to C) The affinity of each *Bp*AXBP toward AXOS or XOS was analyzed by surface plasmon resonance, and their representative sensorgrams are shown. The sensorgrams of all oligosaccharides tested are shown in Fig. S3 in the supplemental material. (D) The affinity of *Bp*AXBP for AXOS or XOS is represented by a heat map based on the calculated *K_d_* value (in micromolar). Each box is colored according to the legend shown on the right. ND^(a)^, no SPR signals were detected; ND^(b)^, the *K_d_* values were too high to be determined. The graphical representations of the glycans are based on reference 38. The stars represent xylose (orange) or arabinose (green).

Previous studies have reported that bifidobacteria possess an arsenal of ABC transporters for the uptake of various carbohydrates (22–24). Among the components of ABC transporters, solute-binding proteins (SBP) play a key role in substrate recognition (25). We prepared three recombinant SBP (denoted B. pseudocatenulatum AXH binding proteins [*Bp*AXBP]) and evaluated their affinity for purified XOS with different degrees of polymerization (from two to six) and AXOS with different arabinose modifications (Fig. S2). We calculated the dissociation constants (*K_d_*) of each *Bp*AXBP for the tested oligosaccharides on the basis of surface plasmon resonance (SPR) (Fig. 2A to C and Fig. S3). Interestingly, we found that three *Bp*AXBP exhibited different substrate specificities (Fig. 2D). *Bp*AXBP3 exhibited low *K_d_* values (less than 100 μM) with all tested oligosaccharides, except for double arabinofuranosyl-decorated XOS (AXOS2), suggesting that the SBP are involved in the uptake of a wide range of AX-derived oligosaccharides. In contrast, the other two *Bp*AXBP displayed narrow and distinct substrate specificities. *Bp*AXBP2 exhibited a high affinity for AXOS, including double arabinofuranosyl-decorated XOS (AXOS2). *Bp*AXBP1 exhibited a high affinity for XOS and AXOS4 (whose nonreducing end of the xylose backbone was not decorated with arabinose) but not for the other AXOS tested.

### Glycoside hydrolase activity of five GH43 enzymes.

**FIG 3:**
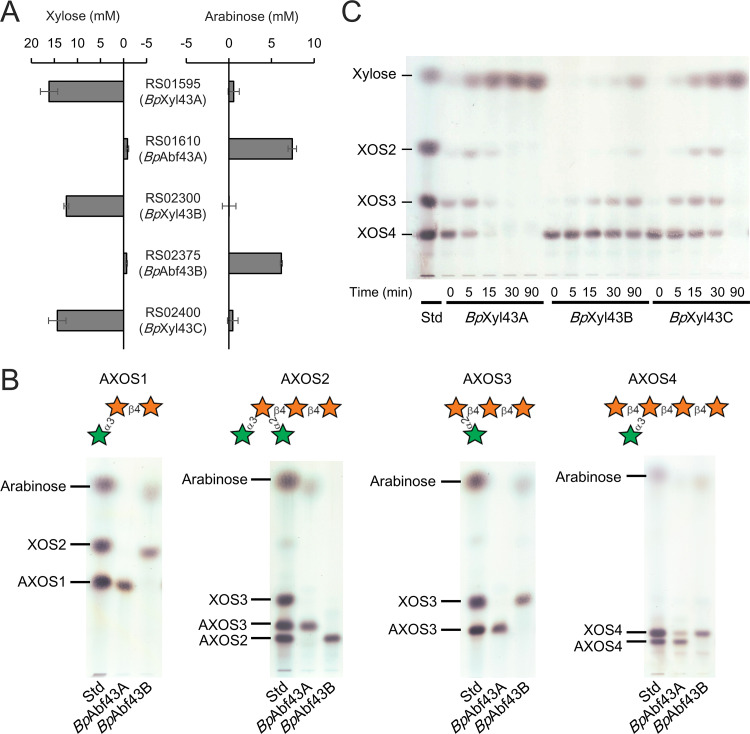
Activity of the five GH43 enzymes toward AX-related oligosaccharides. (A) Concentrations of monosaccharides liberated from AXH by the recombinant GH43 enzymes. The concentration increase from the initial solution is represented. (B) TLC analysis of AXOS by the two recombinant *Bp*Abf43. The substrates are indicated at the top. (C) Time course of hydrolysis of XOS4 by the three recombinant *Bp*Xyl43. The numbers indicate the reaction time of incubation. Lanes Std, a mixture of xylose, XOS2, XOS3, and XOS4. The stars represent xylose (orange) or arabinose (green).

Five GH43 enzymes were upregulated by AXH in our RNA-seq experiment. However, how they cooperatively degraded structurally variable AXH was unclear. Since GH43 enzymes include xylosidases and arabinofuranosidases, we prepared recombinant proteins of the GH43 enzymes and determined their activities by incubating them with AXH. We found that three enzymes (encoded by BBPC_RS01595, BBPC_RS02300, and BBPC_RS02400) liberated xylose, implying that the enzymes were xylosidases (designated *Bp*Xyl43A, *Bp*Xyl43B, and *Bp*Xyl43C) (Fig. 3A). In contrast, the other GH43 enzymes (encoded by BBPC_RS01610 and BBPC_RS02375) liberated arabinose, suggesting that these enzymes were arabinofuranosidases (designated *Bp*Abf43A and *Bp*Abf43B) (Fig. 3A).

### Two arabinofuranosidases exhibited distinct substrate specificities.

We subsequently evaluated the difference in substrate specificities of the two GH43 arabinofuranosidases using purified AXOS with an arabinofuranosyl decoration at the O-2 and/or O-3 position of xylose (Fig. S2B). We found that *Bp*Abf43B was capable of liberating arabinose from AXOS with a single arabinofuranosyl decoration (i.e., AXOS1, AXOS3, and AXOS4); however, the enzyme was incapable of liberating arabinose from AXOS with double arabinofuranosyl decorations (AXOS2) (Fig. 3B). On the contrary, *Bp*Abf43A hydrolyzed the double arabinofuranosyl-decorated AXOS (AXOS2) to a single arabinofuranosyl-decorated AXOS (Fig. 3B); however, there was less or no hydrolyzing activity of the enzyme toward AXOS with a single arabinofuranosyl decoration.

### Three GH43 xylosidases exhibit functionally identical activity.

To confirm the xylosidase activity of the other three GH43 enzymes, we subsequently incubated the recombinant enzymes with XOS with four degrees of polymerization (XOS4). As shown in Fig. 3C, we observed that XOS4 was gradually hydrolyzed and that the amount of xylose was increased in a time-dependent manner, confirming that they had identical xylosidase activity.

### Distribution of the three AXH utilization clusters in other B. pseudocatenulatum strains.

To assess the presence of the three gene clusters among B. pseudocatenulatum strains, we performed BLAST searches against the genomes of 65 strains deposited in the NCBI database (Fig. S1). We found that 13 strains (20%), including the type strain, possessed all three clusters (Fig. S1, group A). Thirty-eight strains (58%, group B) lacked most of the genes located in cluster II, and eight strains (12%, group C) lacked the gene encoded in the former part of cluster I, whereas six strains (9.2%, group D) lacked the genes located in cluster II and the former part of cluster I.

## DISCUSSION

**FIG 4:**
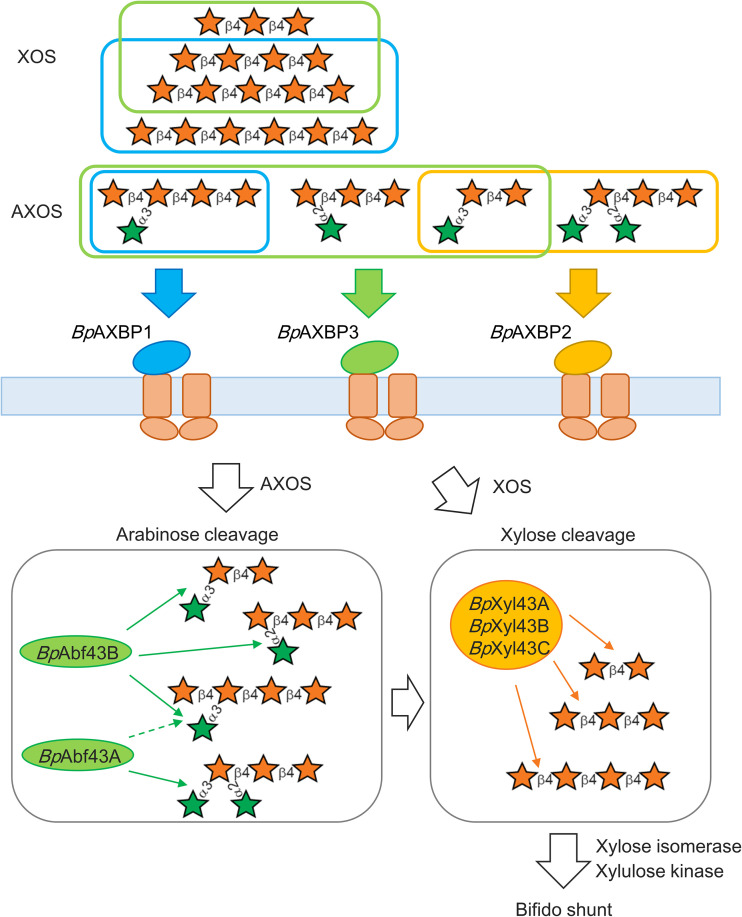
Model of AXH utilization by B. pseudocatenulatum YIT 4072^T^. (Top) Mechanism of AXH uptake; (bottom) model for the degradation of AXH. AXH derived from AX are taken up in the cells by ABC transporters. The lines surrounding the AXOS or XOS indicate that the *K_d_* values were less than 10 μM, and their color corresponds to the color of the *Bp*AXBP. In the bottom part, the arrows from each enzyme indicate the targeted linkages. The broken line from *Bp*Abf43A indicates weak activity compared to that of *Bp*Abf43B. The stars represent xylose (orange) or arabinose (green).

In this study, we found that three ABC transporters, three xylosidases, and two arabinofuranosidases encoded in the three gene clusters were involved in AXH utilization in B. pseudocatenulatum. Three of the *Bp*AXBP had either a broad or a narrow substrate specificity for oligosaccharides, suggesting that they work together to transport AXOS and XOS of different sizes and with different side residue modifications (Fig. 2). Two arabinofuranosidases exhibited distinct substrate specificities, suggesting that they coordinately work to hydrolyze arabinofuranosyl residues decorated at different positions in the xylose backbone (Fig. 3B). We also found that the three xylosidases contributed to hydrolyze the xylose backbone in a similar manner (Fig. 3C). Thus, our study sheds light on the overall picture of how these proteins collaborate for the utilization of structurally diverse AXH in B. pseudocatenulatum YIT 4072^T^, as illustrated in Fig. 4.

### Genes encoded in cluster III may play central roles in AX-OS utilization.

In this study, we found that the ABC transporters and GH43 enzymes encoded in cluster III (i.e., *Bp*AXBP3, *Bp*Xyl43C, and *Bp*Abf43B) exhibited a wide range of substrate specificities and played a major role in AXH utilization. The substrate specificity of these recombinant proteins suggested that most of AXOS and XOS (shown in Fig. S1 in the supplemental material) can be transported and degraded. The broad substrate specificities of the proteins in this gene cluster are in agreement with the fact that cluster III is highly conserved among the B. pseudocatenulatum species.

Consistent with the findings of our study, Ejby et al. reported that an ABC transporter SBP of B. animalis subsp. *lactis* (i.e., *Bl*AXBP), which is a homolog of *Bp*AXBP3 (amino acid homology, 70%), was involved in AXOS and XOS utilization (26). In addition, Andersen et al. reported the involvement of a gene cluster of B. animalis subsp. *lactis* which is similar to gene cluster III involved in XOS utilization found in this study (27).

### ABC transporter and arabinofuranosidase for AXOS with double arabinofuranosyl decoration.

To date, only one bifidobacterial ABC transporter SBP for AXOS and XOS has been characterized (26). However, a transporter for AXOS with a double arabinofuranosyl decoration (AXOS2) (Fig. S2) has not been identified. In this study, we demonstrated an ABC transporter, *Bp*AXBP2, encoded in cluster II, to be responsible for the uptake of AXOS2.

We also found that *Bp*Abf43A, encoded in cluster I, is capable of hydrolyzing the AXOS with a double arabinofuranosyl decoration (AXOS2). The findings of our study are in good agreement with those of a previous study which reported that an arabinofuranosidase AXH-d3 of the other adult-dominant species, B. adolescentis, is capable of hydrolyzing the double-arabinofuranosylated XOS (20).

Of note, the hydrolase and the transporter for the double-arabinofuranosylated XOS are encoded in clusters I and II, respectively. Thus, the difference in substrate specificity of these proteins implies that the genes in these clusters contribute to the complete utilization of AXH with different side residue modifications.

### Bifidobacterial glycoside hydrolases for AXH.

We found that five GH43 enzymes are involved in AXH degradation. Our results are consistent with those of the earlier studies that have reported several bifidobacterial GH43 xylosidases and arabinofuranosidases. Lagaert et al. reported that the xylosidase (XylC) from B. adolescentis LMG 10502^T^ degrades XOS (with a degree of polymerization of from two to six) (21). Viborg et al. reported that BXA43 from Bifidobacterium animalis subsp. *lactis* BB-12 hydrolyzed XOS (with a degree of polymerization of from two to four) (28). As for the arabinofuranosidases, Lagaert et al. reported that AXH-d3 and AbfA from B. adolescentis LMG 10502^T^ cleaved arabinofuranosyl residues from AX (20). The two arabinofuranosidases focused on in this study, *Bp*Abf43A and *Bp*Abf43B, exhibited substrate specificities similar to those of AXH-d3 and AbfA, respectively.

Our study found that GH51 enzymes were not upregulated in B. pseudocatenulatum YIT 4072^T^, although it is known that several GH51 enzymes exhibit arabinofuranosidase activity toward AX (e.g., AbfB from B. adolescentis LMG 10502^T^ and B. longum B667) (20, 29). Our transcriptional analysis indicated that GH43 arabinofuranosidases mainly contributed to AXH degradation in the B. pseudocatenulatum strain.

### Other enzymes in the AXH utilization clusters.

Besides the GH43 enzymes, the results of RNA-seq analysis suggested the involvement of other enzymes in the degradation of AXH (Fig. 1B). We found that the two proteins encoded by BBPC_RS01605 and BBPC_RS02310 are classified as GH8 and that the two proteins encoded by BBPC_RS01600 and BBPC_RS02295 are categorized as GH120, according to the CAZy database (the annotations in the NCBI database and the CAZy assignments are shown in Fig. S1). These enzymes are predicted to be involved in the degradation of the XOS backbone in a manner different from that of GH43 enzymes, as demonstrated for enzymes of Bifidobacterium adolescentis LMG 10502^T^ (21, 30). Our study proposes the mechanism for degradation of the basic structure of AXH; however, further investigation is still needed for a complete understanding of AXH utilization.

### Conclusion.

In this study, we employed RNA-seq analysis to target the genes involved in AXH utilization. Furthermore, we prepared the recombinant enzymes of the target genes to determine whether the transporters and glycoside hydrolases encoded by the three gene clusters work together during the utilization of AXH varying in the length of polymerization and the position of the arabinofuranosyl decoration. Thus, our study contributes to the understanding of how this human gut symbiont became one of the most predominant species in the adult gut.

## MATERIALS AND METHODS

### Oligosaccharides.

Xylobiose (XOS2) was purchased from Tokyo Chemical Industry Co., Ltd. (Tokyo, Japan), and xylotriose (XOS3) was purchased from FUJIFILM Wako Pure Chemical Corporation (Osaka, Japan). The other oligosaccharides used in this study were purchased from Megazyme (Wicklow, Ireland): xylotetraose (XOS4), xylopentaose (XOS5), xylohexaose (XOS6), 3^2^-α-l-arabinofuranosyl-xylobiose (AXOS1), 2^3^,3^3^-di-α-l-arabinofuranosyl-xylotriose (AXOS2), 2^3^-α-l-arabinofuranosyl-xylotriose (AXOS3), and 3^3^-α-l-arabinofuranosyl-xylotetraose (AXOS4). A schematic of these structures is represented in Fig. S2 in the supplemental material. AXH were prepared as follows: 50 ml of 2% (wt/vol) AX (Megazyme) in 100 mM phosphate buffer (pH 6.5) was supplemented with 75 U of endo-xylanase derived from Cellvibrio mixtus (Megazyme); this solution was incubated at 40°C for 16 h and boiled for 10 min to inactivate the enzyme. The boiled solution was added to 200 ml of ethanol, which was then evaporated to obtain the AXH. XOS-95P, which included XOS2 (33.2%), XOS3 (13.78%), and XOS with a degree of polymerization of ≧4 (46.29%) (31), was obtained from B Food Science Co., Ltd. (Aichi, Japan).

### Culture conditions.

B. pseudocatenulatum YIT 4072^T^ was routinely cultured at 37°C in an anaerobic chamber (Coy Laboratory, Grass Lake, MI, USA) with 90% N_2_, 5% CO_2_, and 5% H_2_, using MILS broth (Trypticase peptone, 10 g; yeast extract, 5 g; tryptose, 3 g; K_2_HPO_4_, 3 g; KH_2_PO_4_, 3 g; triammonium citrate, 2 g; pyruvate, 1 ml; cysteine-HCl, 0.3 g; Tween 80, 1 g; MgSO_4_·7H_2_O, 0.575 g; MnSO_4_·4H_2_O, 0.12 g; FeSO_4_·7H_2_O, 0.034 g per liter; pH 6.5) supplemented with 0.5% lactose. For the *in vitro* growth assay, the strain was subcultured in MILS broth supplemented with 0.5% lactose. The cells were washed and suspended in MILS broth without carbohydrates, and aliquots of 2 μl were inoculated into 200 μl of MILS broth supplemented with AX, AXH, XOS-95P, xylose, arabinose, or lactose (final volume, 0.5%). The culture was covered with 50 μl sterile mineral oil to prevent evaporation, and growth was monitored by measuring the optical density at 600 nm (OD_600_) every 30 min using a microplate spectrophotometer (Eon; BioTek, Winooski, VT, USA). The rate of increase in the OD_600_ value from 0.2 to 0.6 was used to calculate the generation time. Lag times were defined as the period until the OD_600_ value reached 0.15.

### Transcriptional analysis.

A culture of B. pseudocatenulatum YIT 4072^T^ in MILS broth supplemented with lactose (0.5%, wt/vol) was washed and resuspended in fresh MILS broth. We inoculated 20 μl of the suspension into 2 ml of MILS broth supplemented with 1% carbohydrates and then incubated the mixture anaerobically at 37°C until the OD_600_ reached 1.0. The bacterial cells were collected by centrifugation (20,000 × *g*, 1 min) and resuspended in RNAlater RNA stabilization reagent (Qiagen, Hilden, Germany) for storage. cDNA libraries were prepared as described in a previous report (32) and sequenced using an Illumina MiSeq instrument with the MiSeq reagent kit (v3) (150 cycles). The reads were filtered as follows: low-quality bases that showed an average quality of less than 30 bp were trimmed off, and reads containing N bases or exhibiting less than 70 bp were removed by using the cutadapt tool (33), before filtering out rRNA reads with the SortMeRNA program (34). The reads obtained were aligned with the genomic sequence of B. pseudocatenulatum YIT 4072^T^ (GenBank accession number NZ_AP012330) by using the Bowtie2 program (35). The amount of reads assigned to each gene was counted by use of the featureCounts program (36). The transcript level was evaluated with the values of the reads per kilobase per million reads (RPKM).

### Cloning, expression, and purification of the recombinant proteins.

The open reading frames (ORF) of the five glycoside hydrolases and the three SBP, excluding the signal peptide sequences predicted by the PSORT server (https://psort.hgc.jp/), were amplified by PCR and cloned into pCold I DNA (TaKaRa Bio Inc., Shiga, Japan), using restriction enzymes (NdeI and XhoI) or an In-fusion HD cloning kit (TaKaRa Bio Inc.). The primers used in this study are listed in Table 1. Each plasmid was transformed into Escherichia coli BL21 (TaKaRa Bio), and the transformants were cultured to express the recombinant N-terminal His-tagged proteins. The culture conditions were according to the manufacturer’s standard protocol, except for E. coli BL21 harboring the *Bp*Xyl43A-expressing plasmid, which was cultured at 15°C after the addition of 1 mM IPTG (isopropyl-β-d-thiogalactopyranoside) and 3% ethanol. The bacterial cells were lysed using 4 ml/g of lysis buffer (B-PER bacterial cell lysis reagent; Thermo Fisher Scientific, Inc., Waltham, MA, USA) containing 100 μg/ml lysozyme and 10 U/ml DNase I, followed by centrifugation at 4°C (15,000 × *g*, 10 min) to obtain the protein fraction. Recombinant proteins were further purified using an Ni-nitrilotriacetic acid spin column (Qiagen) and concentrated by use of a Vivaspin ultrafiltration membrane (GE Healthcare UK Ltd., Little Chalfont, UK). The recombinant proteins prepared were verified using SDS-PAGE (Fig. S4).

**TABLE 1:** Primers used in this study

Target	Primer name-direction^*a*^	Sequence (5′–3′)^*b*^
*Bp*Xyl43A	RS01595-F	CCGGCATATGCAAATCGCAAACCCCGT
	RS01595-R	CCGGCTCGAGCTACTCGTTATCGGGCAATTCC
*Bp*Abf43A	RS01610-F	CCGGCATATGATGATTACCTCAACTAA
	RS01610-R	CCGGCTCGAGTCATTGCTCTCTTTCCTTCG
*Bp*Xyl43B	RS02300-F	CCGGCATATGAAGATCACCAATCCGGT
	RS02300-R	CCGGCTCGAGTCAGTCTTCCATCCCAGAAATT
*Bp*Abf43B	RS02375-F	ATCGAAGGTAGGCATATGACCGCGACTATTACCATTAC
	RS02375-R	AGCAGAGATTACCTACTATGCCATGAAGCCGGC
*Bp*Xyl43C	RS02400-F	CCGGCATATGAAGATTTCCAACCCGGT
	RS02400-R	CCGGCTCGAGCTACTGGTTATCGGAAAGCTCC
*Bp*AXBP1	RS01570-F	ATCGAAGGTAGGCATGCTTGCGGCGGAGGTACTAATA
	RS01570-R	AGCAGAGATTACCTATTACTTCTTAACCTTCAGGTTCTTC
*Bp*AXBP2	RS02280-F	ATCGAAGGTAGGCATGCCAGCAAGGACGAGAATGT
	RS02280-R	AGCAGAGATTACCTATCACTCGGTCGGCAGGGC
*Bp*AXBP3	RS02385-F	ATCGAAGGTAGGCATAAAGACGATAAGACCATTACGTTCTG
	RS02385-R	AGCAGAGATTACCTATCAGCCCTTGGACGCTGC
pColdI	pColdI-F	TAGGTAATCTCTGCTTAAAAGCAC
	pColdI-R	ATGCCTACCTTCGATATGATG

a F, forward; R, reverse.

b The sequences recognizing the restriction enzymes (NdeI or XhoI) are underlined.

### Assay for glycoside hydrolytic activity.

The activity of the five recombinant GH43 enzymes toward AXH was determined by quantifying the liberated xylose or arabinose. A reaction solution containing 2.5% AXH and 0.2 μM enzymes in McIlvaine buffer (pH 6.3) was incubated at 37°C for 16 h, followed by incubation at 94°C for 5 min to inactivate the enzymes. The reaction solution was then mixed with 1 mM glucose as an internal control and analyzed using high-performance liquid chromatography (Shimadzu Corporation, Kyoto, Japan) on a chromatograph equipped with an RI-101 refractive index detector (Showa Denko K.K., Tokyo, Japan) and a KS-802 column (Showa Denko K.K.).

Xylosidase and arabinofuranosidase activities were assessed using purified oligosaccharides. For xylosidase activity, 465 μl of McIlvaine buffer (pH 6.3) mixed with 10 μl of 100 mM XOS4 solution was preincubated at 37°C for 10 min, and then 25 μl of 1.6 μM enzyme was added. After the reaction had started, 50-μl aliquots were collected, heat inactivated at designated time points, and analyzed by thin-layer chromatography (TLC). Arabinofuranosidase activity was examined by analyzing the products in the solution after incubating for 2 h. Reactions were performed in 50 μl of McIlvaine buffer (pH 6.3) with 2 mM AXOS and 0.2 μM enzymes. TLC analysis was performed using TLC silica gel 60 (Merck Millipore, Burlington, MA, USA) and development two times with chloroform-acetic acid-water (6:7:1). Carbohydrates were detected with 0.2% orcinol in sulfuric acid-methanol (1:9) and heating (37).

### Affinity of SBP for XOS and AXOS.

The affinity of each SBP for XOS and AXOS was measured by SPR using a Biacore T200 instrument (GE Healthcare UK Ltd.). Purified recombinant SBP were immobilized on a CM5 sensor chip using an amine coupling kit (GE Healthcare UK Ltd.). As described in a previous report, sensorgrams were obtained at 25°C in 20 mM phosphate/citrate (pH 6.5), 150 mM NaCl, and 0.005% (vol/vol) Tween 20 (Wako) (26). The densities of the immobilized proteins were 13,515 resonance units (RU) (*Bp*AXBP1), 4,354 RU (*Bp*AXBP2), and 3,709 RU (*Bp*AXBP3). Five different XOS and four AXOS were sequentially diluted and applied. The number of resonance units for each concentration was plotted, and *K_d_* values were determined by curve fitting.

### Homology search of B. pseudocatenulatum.

Proteins homologous to the AXH utilization cluster-encoded proteins were obtained using a local BLASTp (v2.7.1) search. The amino acid sequences of the 65 B. pseudocatenulatum strains which were registered with NCBI as of 20 August 2019 were downloaded and analyzed. The BLASTp program was run with an E-value threshold of 10^−5^. Results were filtered by identity (>90%) and coverage (>80%).

### Data availability.

Transcriptome data for B. pseudocatenulatum YIT 4072^T^ during growth in carbohydrates were deposited in the DDBJ Sequence Read Archive (DRA) under BioProject accession number PRJDB10314.

## Supplementary Material

Supplemental file 1
