# Supplementary material for: Multiple Transporters and Glycoside Hydrolases Are Involved in Arabinoxylan-Derived Oligosaccharide Utilization in Bifidobacterium pseudocatenulatum
Source: Appl Environ Microbiol. 2020 Nov 24;86(24):e01782-20. doi: 10.1128/AEM.01782-20 (PMC7688211; doi:10.1128/AEM.01782-20)
Supplement: Supplemental file 1 [file AEM.01782-20-s0001.pdf]

Supplementary information

	locus No.	note	annotation
Cluster I	BBPC_RS01570	*Bp*AXBP1	ABC transporter substrate-binding protein
	BBPC_RS01575		ABC transporter permease
	BBPC_RS01580		ABC transporter permease
	BBPC_RS01585		ABC transporter ATP-binding protein
	BBPC_RS01590		ABC transporter ATP-binding protein
	BBPC_RS01595	*Bp*Xyl43A	glycoside hydrolase family 43 protein
	BBPC_RS01600	GH120	right-handed parallel beta-helix repeat-containing protein
	BBPC_RS01605	GH8	reducing end xylose-releasing exo-oligoxylanase
	BBPC_RS01610	*Bp*Abf43A	glycosyl hydrolase 43 family protein
	BBPC_RS01615	CE10	carboxylesterase family protein
Cluster II	BBPC_RS02280	*Bp*AXBP2	carbohydrate ABC transporter substrate-binding protein
	BBPC_RS02285		sugar ABC transporter permease
	BBPC_RS02290		carbohydrate ABC transporter permease
	BBPC_RS02295	GH120	right-handed parallel beta-helix repeat-containing protein
	BBPC_RS02300	*Bp*Xyl43B	glycoside hydrolase family 43 protein
	BBPC_RS02305		hypothetical protein
	BBPC_RS02310	GH8	xylanase
	BBPC_RS02315		sialate O-acetylesterase
Cluster III	BBPC_RS02370		xylose isomerase
	BBPC_RS02375	*Bp*Abf43B	glycoside hydrolase family 43 protein
	BBPC_RS02380		LacI family transcriptional regulator
	BBPC_RS02385	*Bp*AXBP3	extracellular solute-binding protein
	BBPC_RS02390		sugar ABC transporter permease
	BBPC_RS02395		carbohydrate ABC transporter permease
	BBPC_RS02400	BpXyl43C	glycoside hydrolase family 43 protein
	BBPC_RS02405		9-O-acetylesterase
	BBPC_RS02410		sugar O-acetyltransferase
	BBPC_RS02415		xylulokinase

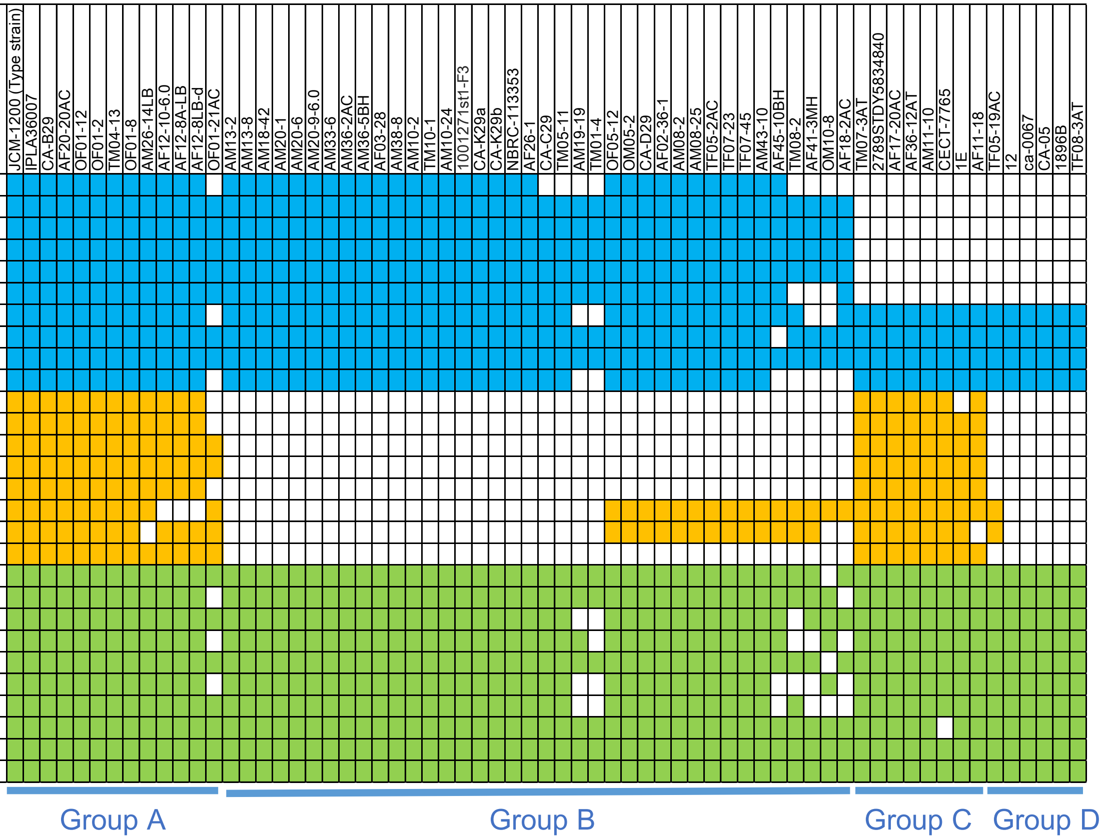

Fig. S1 Conservation of the AXH utilization clusters in 65 *B. pseudocatenulatum* strains.

The presence of the genes in AXH utilization cluster of *B*. *pseudocatenulatum* YIT 4072T was investigated by BLASTp analysis (coverage > 80%, identity > 90% as amino acid sequences) for *B*. *pseudocatenulatum* deposited in the NCBI database. The names of these strains are listed in the top row. Colored squares indicate that the strains possess the homologs. All strains were categorized based on the difference in the presence of the clusters.

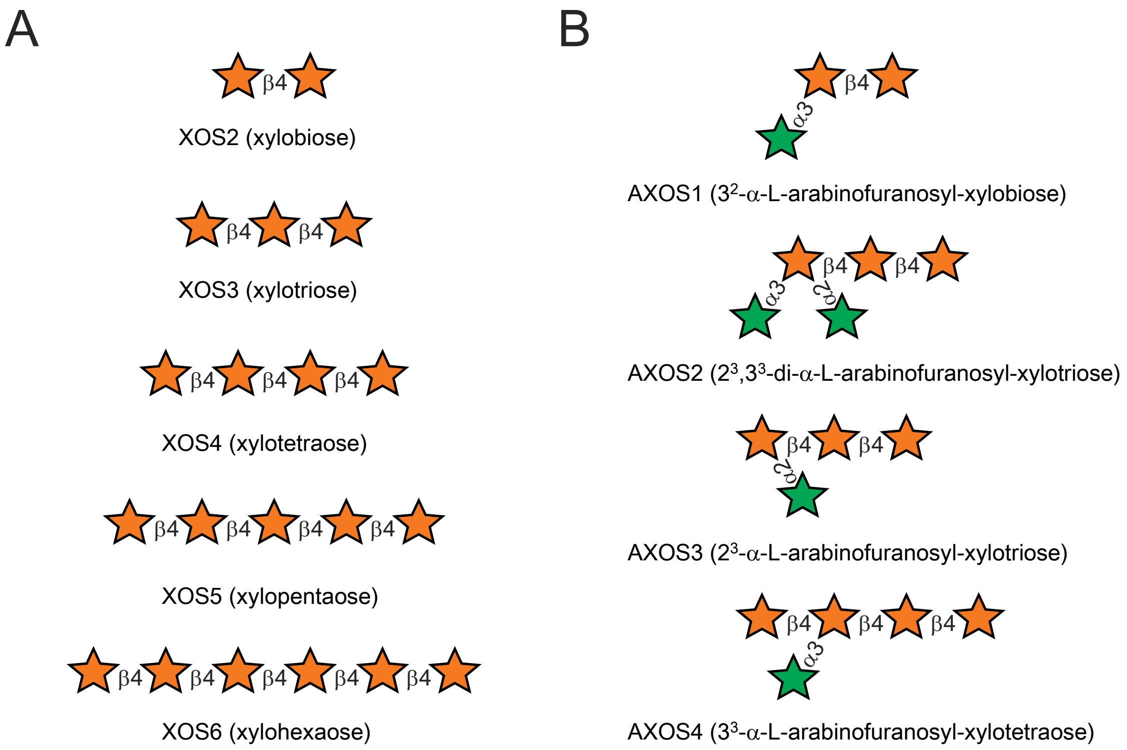

Fig. S2 Structures of XOS and AXOS used in this study.

The structures of (A) XOS and (B) AXOS used in this study. Stars represent xylose (orange) or arabinose (green).

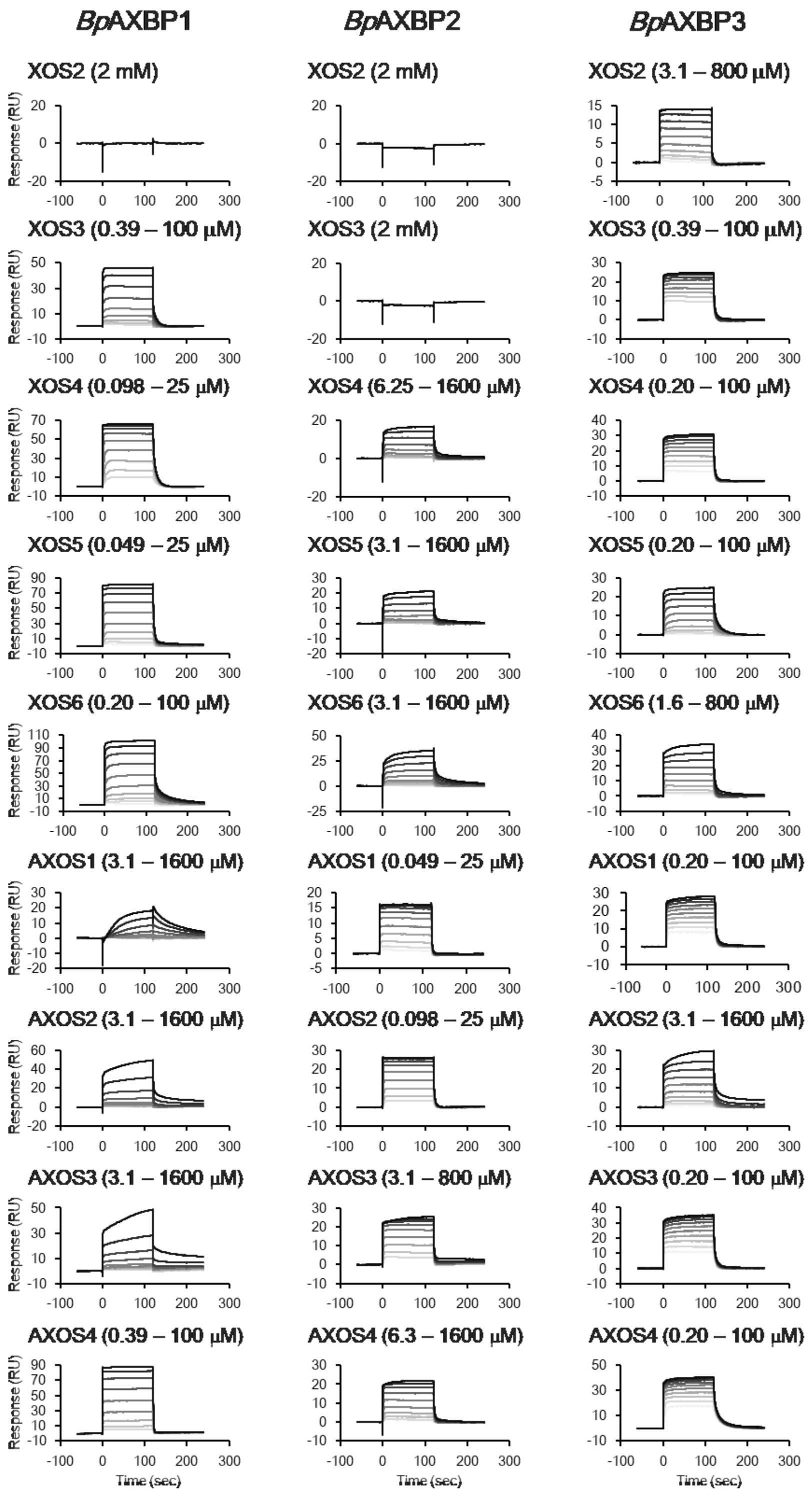

Fig. S3 Affinity of each *Bp*AXBP for AXOS or XOS.

Affinity of each *Bp*AXBP for AXOS or XOS was analyzed by surface plasmon resonance, and their sensorgrams are shown.

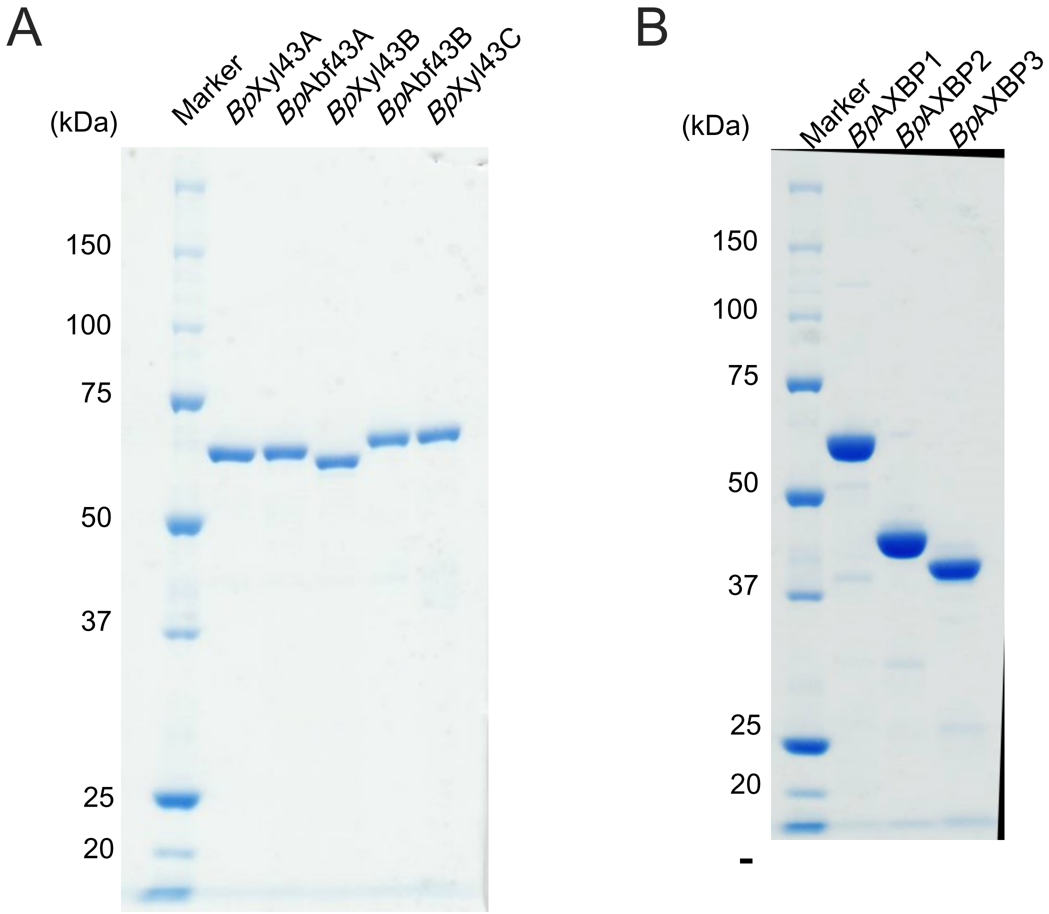

Fig. S4 SDS-PAGE analysis of purified recombinant enzymes.

His-tagged recombinant (A) *Bp*Xyl43, *Bp*Abf43, and (B) *Bp*AXBP were analyzed by SDS-PAGE. Molecular masses of the marker (M) are indicated on the left.
